# Impact of job satisfaction on quality of life in women at the workplace within the cross-sectional Bern Cohort Study 2014 (BeCS-14)

**DOI:** 10.1007/s00404-025-08203-3

**Published:** 2025-10-17

**Authors:** Argyrios Kolokythas, Christina Giese, Michael von Wolff, Norman Bitterlich, Susanne Theis, Sabrina Vollrath, Petra Stute

**Affiliations:** 1 Department of Obstetrics and Gynecology, McGill University Health Centre, Montreal, QC Canada; 2 Department of Obstetrics and Gynecology, Spitalzentrum Biel, Biel, Switzerland; 3 Department of Obstetrics and Gynecology, University Hospital, Inselspital Bern, Friedbuehlstrasse 19, 3010 Bern, Switzerland; 4 Independent Researcher, Draisdorfer Str. 21, 09114 Chemnitz, Germany; 5 Department of Obstetrics and Gynecology, University Medical Center Mainz, Mainz, Germany

**Keywords:** Job satisfaction, Quality of life (QoL), SF-36, IMPULS, Biofunctional status

## Abstract

**Purpose:**

Despite labor laws, over 614 million workers exceed 48 work hours weekly. Excessive work has been linked to health problems, as has job satisfaction which also affects the individual’s quality of life (QoL). This study aims to investigate the impact of job satisfaction on QoL, with a focus on women. Given that occupational stress and job satisfaction have been associated with gynecologic and obstetric outcomes, these findings are of particular relevance to women’s health.

**Methods:**

The study utilized data from the Bern Cohort Study 2014, a single-center, cross-sectional, observational trial. During the study, several background parameters were collected, while the IMPULS questionnaire was used to assess job satisfaction, and biofunctional status (BFS) and SF-36 assessed the QoL. The results were analyzed by non-parametric Spearman–Rho test, parametric Pearson correlation, and ANOVA, controlling for age, income, physical activity, sleep, relationship status, and sexual life.

**Results:**

Our analysis depicted some interesting correlations. For women, an association between their emotional well-being and their energy and fatigue was established with aspects of their job satisfaction, such as variety, completeness and social environment. For men, an obvious association of job control was noted for their general health, energy and fatigue, and emotional well-being. Contrarily, for women job control had no influence on SF-36 parameters but was positively correlated with the systolic blood pressure and diastolic blood pressure (BFS parameters) revealing a rather opposite perception of job control than for men.

**Conclusions:**

Our findings support a link between job satisfaction and QoL. Women consider important factors, such as variety, completeness, and social environment, particularly impacting their emotional well-being and energy levels, whereas for men, job control seems to have the greatest significance. For gynecologists and women’s health providers, recognizing the role of occupational factors may help integrate workplace exposures into patient history-taking and counseling, especially in the context of reproductive and pregnancy health.

## What does this study adds to the clinical work

**Table:** 

This study highlights how job satisfaction and workplace factors affect men and women differently. For obstetrics and gynecology, it emphasizes the importance of considering occupational stress and work environment in patient history, as these can influence quality of life and, consequently, gynecologic health, fertility, and pregnancy outcomes.	

## Introduction

According to data from the International Labor Organization (ILO), despite long-established laws that regulate normal working hours, there are still 22%, or more than 614 million workers worldwide, who work more than 48 h weekly [1]. This phenomenon does not have a geographic pattern, as Peru occupies the first place with 50.9% of people working excessively, with South Korea and Thailand coming second and third with 49.5% and 46.7%, respectively. In Europe, the highest ranked country is the United Kingdom with 25.7% followed by Switzerland with 19.2%. These numbers do not represent a common etiology for this phenomenon either, as in some countries overtime is imposed as an obligation to meet demand, whereas in other countries it is a necessity in order for workers to make ends meet [1].

With those statistics, it becomes obvious that people spend a substantial amount of their everyday lives at their workplace. However, even without excessive working hours, accepting the 40-h-week convention for all individuals, every working person spends approximately 30% of their day at their workplace. Hence, it is inevitable that this time affects other aspects of personal life and health. An association has already been examined between hard data on these two variables, with long working hours being correlated with health problems, such as depression, anxiety, sleep disorders, and coronary artery disease [2]. However, it is not always easy to depict a one-to-one association among all aspects of work life and all aspects of personal life or health, and that is why sometimes a broader categorization in terms of job satisfaction and quality of life (QoL) has been introduced.

Perhaps this is the reason why job satisfaction has been one of the most studied areas of industrial psychology, considered to be an important aspect of the general well-being and life satisfaction of individuals [3]. The first studies on the matter were conducted around 1930 [4], and in fact, nowadays, there is already a plethora of evidence in the literature to suggest that even this broader categorization of job satisfaction is correlated with general health as well as work-related outcomes and behaviors, such as burnout, resignations, and absences from work [5–8]. Job satisfaction has even been suggested as an indicator of psychological well-being [4], while the association between job satisfaction and QoL has been described as a reciprocal causation with one affecting the other and with no causal pathway being able to be demonstrated [9, 10].

Much of the available data concerning job satisfaction comes from studies of healthcare professionals, especially nurses. This data suggests that increased job satisfaction is not only associated with increased QoL but with nursing care too [8]. However, additional data from a German study of 2016 shows a constant decline in job satisfaction over the period from 1990 to 2012 [11], while several other studies also depict a low level of job satisfaction among nurses [6, 12]. Knowing that there is an association between job satisfaction and QoL and having evidence to support that the former is in decline over the years, at least as far as nurses are concerned, generates a need to investigate similar associations between job satisfaction and QoL in the general population too, since this could have a greater impact on population health.

In addition, as much of the evidence comes from nursing, a field, where the majority of the workforce is women, these findings can, with some limitations, be extrapolated to women’s health more broadly. The relevance of job satisfaction and work environment, therefore, extends directly to Obstetrics and Gynecology. Occupational stress, rotating shift work, extended working hours, and low job satisfaction have indeed already been linked to multiple gynecologic conditions and obstetrical complications, including menstrual irregularities, infertility, miscarriages, preterm birth, and hypertensive disorders of pregnancy [13–15]. Although frequently overlooked in clinical settings, consideration of occupational factors by gynecologists and women’s health providers should be essential for comprehensive patient care.

The aim of this study is to evaluate the impact of job satisfaction, measured using the IMPULS questionnaire [16] on the QoL as assessed by both SF-36 [17, 18], a validated but subjective rating tool as well as by biofunctional status (BFS) assessment tool [19, 20], which uses objective measurements in physical, mental-cognitive, emotional, and social domains, and whose results have recently been correlated with those of SF-36 in an attempt to objectify QoL measurements [21].

## Methods

### Study design and population

To answer our research question, we used data from the Bern Cohort Study 2014 (BeCS-14) [22], a single-center, cross-sectional, observational, non-interventional, non-randomized trial conducted between March and July 2014 at the Department of Obstetrics and Gynecology of Inselspital of the University of Bern in Switzerland. The cohort included a German-speaking adult population up to the age of 65 years. Several parameters were collected at the time of the study, during an 80-min interview, among which personal and family history, parameters needed to assess the BFS as well as the health-related QoL through the SF-36 questionnaire [17]. For participants generating income through regular jobs, additional information was collected by giving them 10 min to answer the IMPULS questionnaire, assessing real and favored job life situations.

### Assessment of job satisfaction (IMPULS questionnaire)

To assess job satisfaction, we used the IMPULS questionnaire [16]. IMPULS is a 25-question risk assessment tool concerning psychosocial stressors in the workplace, assessing an individual’s work situation. Each of the questions needs to be answered twice, first addressing the real work environment and a second time for the ideal job. The 25 items then form 11 subcategories that merge into 5 main categories [23]. Details of all categories and subcategories as well as the items assessed in each can be found in Table 1.

**Table 1:**
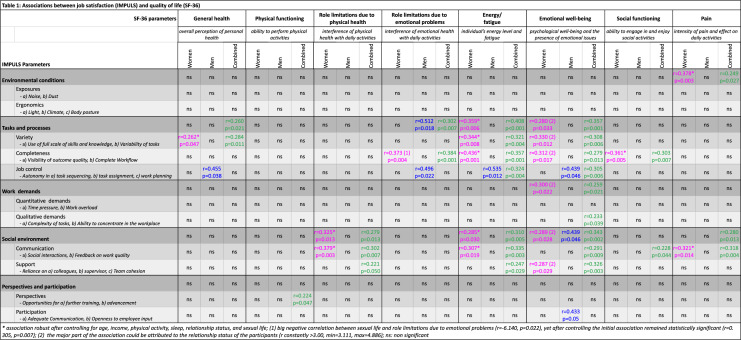
Associations between job satisfaction (IMPULS) and quality of life (SF-36)

### Assessment of quality of life (SF-36 questionnaire)

For the assessment of the QoL, we used the SF-36 questionnaire. The SF-36 is, since 1992, a well-established, widely used, and easily administered, self-reporting 36-item tool with generic QoL measures categorized into 8 health concepts [17, 18], which can be found in Table 1.

### Assessment of the biofunctional status (BFS) and biofunctional age (BFA)

The BFS is a comprehensive and validated age- and sex-specific tool test developed by Poethig et al. [19, 20], (commercially available via vital.services) and which assesses individuals’ characteristics with a holistic approach from many different aspects, including physical, psychological/emotional, and social characteristics. The summation of BFS' results is used in conjunction with gerontologic aging tables to calculate the BFA. Detailed information about the conversion of BFS to BFA has been previously published [24].

### Statistical analysis

To investigate the correlations between IMPULS and the different QoL measures (SF-36, BFS), we used the non-parametric Spearman–Rho test. The reported results about correlations are those of the Spearman–Rho test (coefficient r, *p* value). We also used the parametric Pearson correlation and ANOVA for our analysis as a linear regression model in an attempt to quantify the magnitude of any potential correlation. Prior to applying parametric tests, we assessed the normality of the variables using Kolmogorov–Smirnov and Shapiro–Wilk tests. The analyses were based on established continuous scores from the SF-36, IMPULS, and BFS/BFA instruments, which are appropriate for parametric methods. This analysis was performed only for women participants as the identified sex-specific differences necessitated separating participants by sex and the limited number of men participants did not allow for such an analysis in that group. We believe that the analysis we performed for the women is robust even in the absence of normal distribution of the variables. For all statistical tests, a two-tailed alpha level of 0.05 was used to indicate significance. When enough power could be guaranteed, we also controlled for age, income, physical activity, sleep, relationship status, and sexual life.

## Results

In this study, we had 79 participants, 58 women, and 21 men. The median age of the whole cohort was 36 years (range 22–68 years), while for women, it was 38 years and for men 28 years. In the cohort participated 10 students (7 women, 3 men) and 2 trainees (1 woman, 1 man), while the rest were employed individuals, of whom 11 women (19.3%) and 5 men (23.8%) held leading positions. The majority of the participants (50% of women and 52.4% of men) reported a monthly income between 5,000 and 10,000 Swiss francs, and 1.7% of women and 14.3% of men exceeded 10,000 Swiss francs monthly.

Due to BeCS-14’s recruiting process (organized by the Department of Obstetrics and Gynecology), the main focus of data collection was women; however, we had some limited datapoints from male participants, which we used when statistically allowed. Despite the availability of several pieces of background information, the small number of participants limited our ability to control for all variables. When allowed, we controlled for the aforementioned variables, as described in our methods section.

### Association of IMPULS and SF-36

We first analyzed our data using the health concepts of the SF-36 questionnaire as the dependent variable. The detailed results are presented in Table 1. For women, we found that all but one (physical functioning) out of the eight SF-36 health concepts were associated with IMPULS, and from a different perspective, all but one (perspectives and participation) out of the five IMPULS categories were associated with SF-36. Women's general health was positively associated with variety from the tasks and processes category (*r* = 0.262, *p* = 0.047), while social functioning was associated with completeness (*r* = 0.361, *p* = 0.005) from the same IMPULS category. Completeness was also associated with role limitations due to emotional problems (*r* = 0.373, *p* = 0.004), an association that remained statistically significant (*r* = 0.305, *p* = 0.007), despite a big negative correlation between sexual life and role limitations due to emotional problems (β = -6.140, *p* = 0.022) that was noted when controlling for the initial association. Role limitations due to physical health were associated with social environment (*r* = 0.325, *p* = 0.013), and the subcategory communication (*r* = 0.379, *p* = 0.003) separately. The latter, as a subcategory solely, was also associated with pain (*r* = 0.321, *p* = 0.014), as were the environmental conditions (*r* = 0.378, *p* = 0.003).

Energy and fatigue, together with emotional well-being, revealed the majority of associations. The first was positively associated with tasks and processes (*r* = 0.359, *p* = 0.006) as well as with the subcategories variety (*r* = 0.344, *p* = 0.008) and completeness (*r* = 0.436, *p* = 0.001) separately. Furthermore, an association between social environment (*r* = 0.285, *p* = 0.030) and the subcategory communication (*r* = 0.307, *p* = 0.019) was noted. Emotional well-being was also associated with tasks and processes (*r* = 0.280, *p* = 0.033), variety (*r* = 0.330, *p* = 0.012), and completeness (r = 0.312, *p* = 0.017) as well as with social environment (*r* = 0.289, *p* = 0.028) and the subcategory support (*r* = 0.287, *p* = 0.029), and was the only one to show an association with work demands too (*r* = 0.300, *p* = 0.022). Yet, for the whole health concept of emotional well-being, the major part of the association could probably be attributed to the relationship status of the participants, as after controlling, a substantial and stable positive association was revealed (β all larger than 3.00, min = 3.111, max = 4.886).

When separately examining the male participants, only four out of the eight SF-36 health concepts were affected by the IMPULS parameters. Job control, from tasks and processes, had a positive association with general health (*r* = 0.455, *p* = 0.038), energy and fatigue (*r* = 0.535, *p* = 0.012), role limitations due to emotional problems (*r* = 0.496, *p* = 0.022), which was also associated with the whole category of tasks and processes separately (*r* = 0.512, *p* = 0.018), and finally, emotional well-being (*r* = 0.439, *p* = 0.046). The latter revealed also an association with social environment (*r* = 0.439, *p* = 0.046) and also a marginal association with participation (*r* = 0.433, *p* = 0.05). The results for the combined female and male group can be found in Table 1.

To study whether the difference between the actual and the ideal working conditions, representing missing expectations (i.e., a negative perception of the current situation in comparison to the ideal situation one would imagine possible), had an impact on the QoL, we ran an analysis (only for the female participants) on this difference and the SF-36, with some interesting results. Those results, after controlling for the actual work situation and for age, income, physical activity, sleep, relationship status, and sexual life, are presented in Table 2.

**Table 2:**
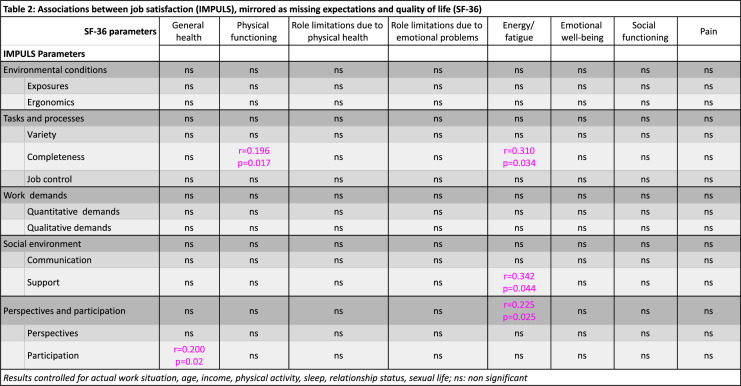
Associations between job satisfaction (IMPULS), mirrored as missing expectations and quality of life (SF-36)

Interestingly, it was again energy and fatigue that yielded the most associations, those being with completeness (*r* = 0.310, *p* = 0.034) from the task and processes category, support (*r* = 0.342, *p* = 0.044) from the social environment category, and the whole perspectives and participation category (*r* = 0.225, *p* = 0.025). The two other SF-36 concepts that revealed associations were physical functioning with an association with completeness (*r* = 0.196, *p* = 0.017), and general health with participation (*r* = 0.200, *p* = 0.020).

### Association of IMPULS and BFS/BFA

No correlation between BFA and job satisfaction was observed. When we analyzed the BFS categories, though, and the retrieved individual values, we found some interesting correlations. We focused the analysis for BFS only on the women participants (Table 3).

**Table 3:**
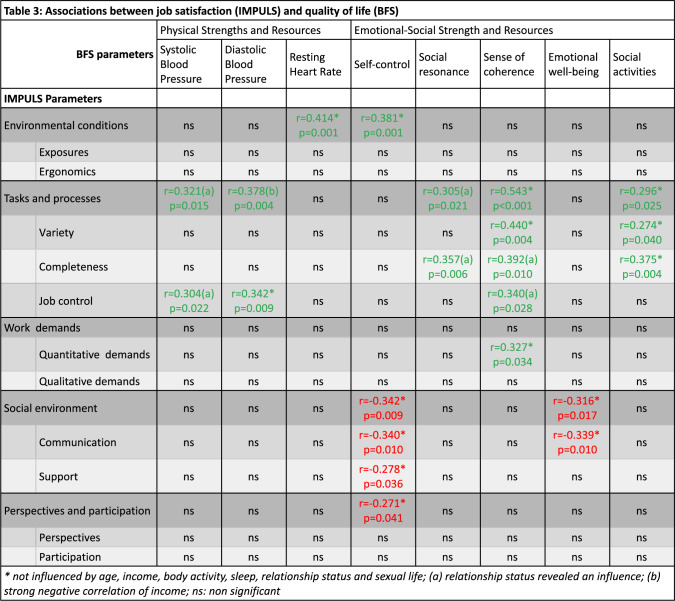
Associations between job satisfaction (IMPULS) and quality of life (BFS)

From all BFS categories, only physical strengths and resources and emotional–social strength and resources yielded associations with the IMPULS parameters. From the first category, both systolic (SBP) and diastolic (DBP) blood pressure were associated with tasks and processes (*r* = 0.321, *p* = 0.015 and *r* = 0.378, p = 0.004, respectively), and the subcategory job control (*r* = 0.304, *p* = 0.022 and *r* = 0.342, *p* = 0.009, respectively), while heart rate was associated with environmental conditions (*r* = 0.414, *p* = 0.001).

Tasks and processes were also associated with social resonance (*r* = 0.305, *p* = 0.021), social activities (*r* = 0.296, *p* = 0.025) and sense of coherence (*r* = 0.543, *p* < 0.001) as was the subcategory completeness (*r* = 0.357, *p* = 0.006; *r* = 0.375, *p* = 0.004; *r* = 0.392, *p* = 0.010, respectively). Sense of coherence was additionally associated with variety (*r* = 0.440, *p* = 0.004), job control (*r* = 0.340, *p* = 0.028) and quantitative demands (*r* = 0.327, p = 0.034). In many of the aforementioned associations, however, the relationship status had a big influence too. For the last two BFS categories to have shown associations with IMPULS, self-control, and emotional well-being, several additional associations were noted, the majority of which were negative. Self-control was positively associated with environmental conditions (*r* = 0.381, *p* = 0.001) and negatively associated with perspectives and participation (*r* = -0.271, *p* = 0.041) as well as social environment (*r* = -0.342, *p* = 0.009) and its two subcategories: communication (*r* = -0.340, *p* = 0.010) and support (*r* = -0.278, *p* = 0.036). Finally, emotional well-being was negatively associated with social environment (*r* = -0.316, *p* = 0.017) and communication (*r* = -0.339, *p* = 0.010) too.

## Discussion

In line with current literature, the results of this study depict an association between job satisfaction and QoL and suggest some patterns in the importance of specific aspects of job satisfaction as those interpreted by the IMPULS questionnaire on specific aspects of QoL. Although research has shown work environment [25, 26] and performance [27] to be some of the most important drivers of job satisfaction, this study depicted different variables of the workplace associated with QoL. From the perspective of women’s health, these findings are particularly relevant, as occupational well-being is increasingly recognized as a determinant of gynecologic and obstetric outcomes, such as menstrual regularities, fertility, and pregnancy complications [13–15]. That is probably the reason why both the American College of Obstetricians and Gynecologists (ACOG) and the American Society for Reproductive Medicine (ASRM) emphasize the need to assess occupational exposures during preconception counseling and prenatal care [28].

Our first analysis using the SF-36 questionnaire yielded some interesting results. It appears that for women, the major effect of job satisfaction was depicted in the emotional well-being as well as the energy and fatigue categories, with variety and completeness as well as social environment affecting both of these parameters. From a different perspective, completeness was the IMPULS parameter that had the highest association with four aspects of SF-36 QoL (role limitations due to emotional problems, social functioning, energy and fatigue, and emotional well-being), and variety came second with 3 (energy and fatigue, emotional well-being, and general health). Job control was the only subcategory of “tasks and processes” not to show any associations for the women in the study. In addition to energy and fatigue and emotional well-being, social environment, and more specifically, communication showed an effect on role limitations due to physical health and pain. From these results, it appears that, at least for women, the job parameters that are considered more important and can potentially influence their general QoL are mostly the social characteristics of their job environment, whereas other more rigid parameters, such as the physical environment and work demands, did not appear to have an effect on their satisfaction.

Indeed, both variety [29] and completeness [30] have also been previously correlated with both job and life satisfaction. Although the former is more studied, it appears to be more dependent on the individual’s personality, as discussed by Cueto and Pruneda, with individuals who like a variety of tasks in their workplace perceiving that as a positive aspect, while others might find it a source of stress and dissatisfaction. The data for the latter are more limited and mostly indirect, suggesting a triple-way connection between completeness, lack of support, and life satisfaction. From the category of social environment, communication has been studied a lot and has been shown to be constantly positively correlated with satisfaction both within the workplace but also life satisfaction in general [31, 32].

The above findings align with prior evidence that social support and communication are critical parameters in women’s health, reducing stress-related gynecologic conditions, improving pregnancy outcomes [33], and lowering rates of postpartum depression [34]. These aspects hold broader clinical significance for women’s health providers, emphasizing the need to address workplace dynamics as part of preventive care. At the same time, the physical demands of work should not be overlooked: prolonged standing and repetitive lifting have been linked to adverse outcomes, such as miscarriage, preterm birth, low birthweight, and preeclampsia [35, 36]. Although physical stressors were not strongly reflected in this study, this may suggest that women do not always consciously perceive them as harmful, even though evidence consistently shows their association with adverse outcomes. In addition, organizational stressors—including low decision latitude, irregular shifts, night work, and other psychosocial factors—have been associated with modestly increased risks of pregnancy loss and fetal growth restriction [35, 37, 38], further highlighting the intersection between psychosocial and biological pathways.

Contrary, for men, it was job control that showed the most associations with different parameters of SF-36 QoL (general health, role limitations due to emotional problems, energy and fatigue, emotional well-being), while environment and participation seemed to be associated with emotional well-being too. Although, as mentioned before the sample for men was very limited and no safe conclusions can be drawn, previous research also supports the evidence that influence over their work is more important for men [39], while is also positively correlated with the individual’s well-being [40]. Nordenmark et al., however, found a positive correlation in both female and male employees, but in self-employed individuals, this was only true for men [40]. There is hence a clear indication that job control is an important parameter of job satisfaction that can affect the QoL, more so for men, which might indicate some social stereotypes still in place with men needing to have more influence in their workplace. For gynecologists, these gender differences may parallel broader patterns of health-seeking behavior, coping strategies, and stress responses between men and women, emphasizing the need for tailored workplace and health interventions.

Interestingly, when we studied whether the difference between the actual and the ideal working conditions had an impact on QoL for women, completeness retained its significance as a general idea affecting energy and fatigue, revealing also a new correlation with physical functioning. Another new correlation that was not picked up by our first analysis appeared between participation and both general health and energy and fatigue categories. This might be perceived as a need of the employees for more participation and shared decisions in an environment, where their voices are heard. For women, greater participation and shared decision-making in the workplace may mirror and reinforce their sense of autonomy in reproductive and health-related decisions, suggesting that empowerment at work and empowerment in personal health are closely interconnected.

From the second analysis that we performed using BFS, we found interesting results too. As we discussed above, job control did not seem an important variable for women when answering the questionnaires, yet physical evidence revealed an association between job control and both systolic and diastolic blood pressure. In addition, in spite of environmental conditions not being highlighted when answering the questionnaires, an association was noted between those and the resting heart rate. These physiological findings strengthen the evidence that occupational factors exert measurable effects on women’s health—beyond subjective perception—by influencing cardiovascular function and adaptation in pregnancy and potentially contributing to long-term risk of hypertensive disorders [41]. Moreover, recent data suggest that when physical stressors, such as postural constraints overlap with psychosocial stressors, they can further exacerbate risks like fetal growth restriction [37], underscoring the multi-dimensional impact of workplace exposures.

Taken together, our findings add to the growing literature linking job satisfaction with women’s reproductive and overall health. For clinicians in Obstetrics and Gynecology, understanding these associations may facilitate more comprehensive history-taking, preventive counseling, and interdisciplinary collaboration with occupational health specialists. Moreover, professional societies such as ACOG recommend risk mitigation through workplace accommodations and exposure reduction for pregnant workers [28], reinforcing the clinical responsibility to integrate occupational health into women’s healthcare.

This study is not without its limitations. First of all, the number of participants cannot allow for generalizability of the results. Moreover, as a cross-sectional observational study, it is prone to selection bias, especially taking into consideration the lack of randomization and the fact that the recruitment was performed among individuals in the proximity of the Inselspital in Bern. In addition, and despite the use of standardized questionnaires with overall proven reliability, information bias cannot be excluded, while the lack of longitudinal follow-up prevents conclusions on causality and on how job satisfaction trajectories may influence long-term reproductive and obstetric outcomes. Future studies should aim to integrate both psychosocial and physical exposures in larger, longitudinal cohorts. Nevertheless, this study seems to support the current literature about the association between job satisfaction and QoL and is the first to do so using the IMPULS questionnaire.

## Conclusion

To conclude, our results, in line with current literature, suggest an association between job satisfaction and QoL. Women, consciously or subconsciously, seem to consider variety, completeness, and communication in the workplace as the most important parameters that can potentially affect their QoL and mostly their emotional well-being and energy and fatigue levels. Despite the limited number of participants, job control steadily seemed to be important for the male participants only. From an obstetrics and gynecology perspective, these findings are especially relevant, as occupational well-being has been linked not only to general quality of life but also to reproductive health outcomes, pregnancy complications, and long-term cardiovascular risk. Integrating occupational history and workplace exposures into routine gynecologic and prenatal care could, therefore, help clinicians identify women at higher risk, guide counseling, and support workplace accommodations that promote both maternal health and favorable pregnancy outcomes.

## Data Availability

No data sets were generated or analyzed during the current study.
